# Supplementary material for: Relative influence of intellectual disabilities and autism on mental and general health in Scotland: a cross-sectional study of a whole country of 5.3 million children and adults
Source: BMJ Open. 2019 Aug 27;9(8):e029040. doi: 10.1136/bmjopen-2019-029040 (PMC6719834; doi:10.1136/bmjopen-2019-029040)
Supplement: Author's manuscript [file bmjopen-2019-029040.draft_revisions.pdf]

BMJ Open

BMJ Open is committed to open peer review. As part of this commitment we make the peer review history of every article we publish publicly available.

When an article is published we post the peer reviewers' comments and the authors' responses online. We also post the versions of the paper that were used during peer review. These are the versions that the peer review comments apply to.

The versions of the paper that follow are the versions that were submitted during the peer review process. They are not the versions of record or the final published versions. They should not be cited or distributed as the published version of this manuscript.

BMJ Open is an open access journal and the full, final, typeset and author-corrected version of record of the manuscript is available on our site with no access controls, subscription charges or pay-per-view fees (http://bmjopen.bmj.com).

If you have any questions on BMJ Open's open peer review process please email info.bmjopen@bmj.com

BMJ Open

The relative influence of intellectual disabilities and autism on mental and general health. A cross-sectional study of a whole country of 5.3 million children and adults

Journal:	*BMJ Open*
Manuscript ID	bmjopen-2019-029040
Article Type:	Research
Date Submitted by the Author:	09-Jan-2019
Complete List of Authors:	Kinnear, Deborah; University of Glasgow, Institute of Health and Wellbeing<br>Rydzewska, Ewelina; University of Glasgow, Institute of Health and Wellbeing<br>Dunn, Kirsty; University of Glasgow, Institute of Health and Wellbeing<br>Hughes-McCormack, Laura; University of Glasgow, Institute of Health and Wellbeing<br>Melville, Craig; University of Glasgow<br>Henderson, Angela; University of Glasgow, Institute of Health and Wellbeing<br>Cooper, Sally-Ann; Glasgow University , Institute of Health and Wellbeing
Keywords:	intellectual disabilities, autism, MENTAL HEALTH, general health, health inequalities

SCHOLARONE™
Manuscripts

The relative influence of intellectual disabilities and autism on mental and general health. A cross-sectional study of a whole country of 5.3 million children and adults

***Deborah Kinnear[1] PhD**
Ewelina Rydzewska[1] PhD
Kirsty Dunn[1] MA
Laura Hughes-McCormack[1] MA
Craig Melville[1], MD FRCPsych
Angela Henderson[1] BSc
Sally-Ann Cooper[1] MD FRCPsych

*Correspondence: Institute of Health and Wellbeing, University of Glasgow, Mental Health and Wellbeing Group, 1st Floor Administrative Building, Gartnavel Royal Hospital, 1055 Great Western Road, Glasgow G12 0XH, UK
Deborah.Kinnear@glasgow.ac.uk
[1]Mental Health and Wellbeing Research Group, Institute of Health and Wellbeing, University of Glasgow, Glasgow, UK

Word Count: 2,988

ABSTRACT

Objectives: To determine the relative extent that autism and intellectual disabilities are independently associated with poor mental and general health, in children and adults.
Design: Cross-sectional study. For Scotland's population, logistic regressions investigated odds of intellectual disabilities and autism predicting mental health conditions, and poor general health, adjusted for age and gender.
Participants: 1,548,819 children/youth aged 0-24y, and 3,746,584 adults aged 25y+, of whom 9,396/1,548,819 children/youth had intellectual disabilities, 25,063/1,548,819 children/youth had autism; and 16,953/3,746,584 adults had intellectual disabilities, 6,649/3,746,584 adults had autism.
Main outcome measures: General health status and mental health
Results: In children/youth, intellectual disabilities (OR=7.04; CI 6.30, 7.87) and autism (OR=25.08, CI 23.08, 27.32) both independently predicted mental health conditions. In adults, intellectual disabilities (OR=3.50; CI 3.20, 3.84), and autism (OR=5.30, CI 4.80, 5.85) both independently predicted mental health conditions. In children/youth, intellectual disabilities (OR=18.34, CI 17.17, 19.58) and autism (OR=8.40, CI 8.02, 8.80) both independently predicted poor general health. In adults, intellectual disabilities (OR=7.54, CI 7.02, 8.10), and autism (OR=4.46, CI 4.06, .89) both independently predicted poor general health.
Conclusions: Both intellectual disabilities and autism independently predict poor health, intellectual disabilities more so for general health, and autism more so for mental health. Intellectual disabilities and autism are not uncommon, and due to their associated poor health, sufficient services/supports are needed. This is not just due to co-existence of these conditions, or just to having intellectual disabilities, as autism also is independently associated with substantial health inequalities across the entire life-course

Key words: intellectual disabilities, autism, mental health, general health, health inequalities

For peer review only - http://bmjopen.bmj.com/site/about/guidelines.xhtml

Strengths and limitations of this study

- This large study is the first study to have reported on the extent to which autism and intellectual disabilities are independently associated with poor mental and general health, in children and adults.
- The study comprises a whole country population, with high participation rate (94%), and the conditions were systematically enquired about on everyone.
- A limitation is that conditions were self/proxy reports rather than in-depth diagnostic assessments.

For peer review only

INTRODUCTION

Both intellectual disabilities and autism occur not uncommonly in children and adults, and can co-occur. Children and adults with intellectual disabilities have notably poorer mental and general health than other people,[1-4] and this has also been reported for autistic children and adults,[5-9] although the quantity of research is limited, particularly with regards to adults. The extent of co-occurrence of intellectual disabilities in autistic people used to be considered to be as high as 50-70%,[10] although more recent reports suggest it may be lower, though still considerable, at about 20%.[9,11] This may in part relate to the broadening of criteria for the autism spectrum to include "milder" autism and greater awareness about autism in children and young people in recent years, as it is well established that autism prevalence is higher in people with more severe intellectual disabilities and vice-versa.[12] Whilst autism is generally considered to be associated with poor mental health, the largest study to examine this in adults in a general community population found no difference in rates of mental ill-health in adults with co-occurring autism and intellectual disabilities compared with age-gender-Down syndrome-level of ability-matched adults with intellectual disabilities but no autism.[13] As intellectual disabilities and autism have tended to be studied separately, the relative extent to which being autistic, or having intellectual disabilities, accounts for their poor population health is not clear. This is important to understand, given the frequent co-occurrence of these conditions, and the more recent change in co-occurrence due to higher frequency of diagnosis of autism.

The aim of this paper is to study the extent to which autism and intellectual disabilities are independently associated with poor mental and general health, in children and adults.

METHOD

Approval

Approval was gained from the Scottish Government for secondary analysis of the Scotland Census, 2011 data, and access to a subset of data was provided.

Census process and variables

Scotland has performed a national Census every 10 years since 1841, the most recent being Scotland's Census, 2011. Information was collected on every resident in Scotland on the Census date, 27.3.11. This included people in private households and also people in

For peer review only - http://bmjopen.bmj.com/site/about/guidelines.xhtml

community residences (such as care homes, prisons, student halls of residence). In private households (typically family households), one person was responsible for completing the census details for all the household's residents; for communal establishments the manager was responsible for providing the information. Failure to provide information, or for providing false information attracted a fine of up to £1,000. Non-responses were followed up by the Census team and help provided. These factors accounted for the high response rate; Scotland's Census 2011 achieved a 94% response rate.[14] The Census team adjusted for the 6% non-response rate using a Census Coverage Survey to estimate numbers and characteristics. The Census Coverage Survey included around 40,000 households; the records from it were matched with Census records, with all individuals deterministically matched to check for duplicates. Individuals estimated to be missing from the Census were then imputed, using a subset of characteristics from real individuals, including health information. This Edit and Imputation Methodology was adapted from the Office for National Statistics rigorous and systematic guidelines, available at:
http://webarchive.nationalarchives.gov.uk/20160108193745/http://www.ons.gov.uk/ons/guide-method/method-quality/survey-methodology-bulletin/smb-69/index.html
and further details on the Census population estimates are available at:
http://www.scotlandscensus.gov.uk/documents/censusresults/release1b/rel1bmethodology.pdf
Full details of the methodology and other background information on Scotland's Census, 2011 are available at:
http://www.scotlandscensus.gov.uk/supporting-information.

The Census included questions on demography, long-term conditions, and on general health.

The question on long-term conditions enquired:
"Do you have any of the following conditions which have lasted, or are expected to last, at least 12 months? Tick all that apply:

- Deafness or partial hearing loss
- Blindness or partial sight loss
- Learning disability (for example, Down's Syndrome)
- Learning difficulty (for example, dyslexia)
- Developmental disorder (for example, Autistic Spectrum Disorder or Asperger's Syndrome)
- Physical disability
- Mental health condition
- Long-term illness, disease or condition
- Other condition, please write in
    - free text space was then provided for conditions to be listed

or

- No condition"

The question on general health enquired:
"How is your health in general?

- Very good
- Good
- Fair
- Bad
- Very bad"

For peer review only - http://bmjopen.bmj.com/site/about/guidelines.xhtml

The terminology used in both these questions was specifically investigated prior to implementation of data collection. The General Register Office for Scotland commissioned Ipsos MORI Scotland to undertake cognitive question testing, to determine whether the questions were answered accurately and willingly by respondents, and what changes if any might be required to improve data quality and/or the acceptability of the response options. Cognitive interviewing is a widely used approach to critically evaluate survey questionnaires.[15] It enables researchers to modify survey material to enhance clarity. Retrospective probing was deemed to be the most appropriate of the different techniques available. This research was undertaken with 102 participants with a mix of gender and age, both with and without the health conditions and disabilities (including people with more than one of the conditions). This included people with autism, intellectual disabilities, dyslexia, dyspraxia, speech impairment, mental health conditions (both milder and more serious), and other long-term conditions. The results found that the question on general health status functioned well and did not need amendment, whilst the question on autism was redesigned to that listed above in order to more accurately capture the data specifically on autism. Additionally, the response "no" was amended to "no condition". The other questions did not require any modification. Further information can be found at:
http://www.scotlandscensus.gov.uk/documents/research/2011-census-health-disability-questions.pdf
http://www.scotlandscensus.gov.uk/documents/legislation/changes-to-gov-statement-report.pdf

In Scotland, the term "learning disability" is synonymous with the international term "intellectual disabilities".

For 2.6% of the Census returns, information on long-term conditions was not completed. The Census team assumed the most plausible explanation was that the person had no long-term condition but did not see the 'No condition' check box at the end of the question, and hence recorded them to have none of the long-term conditions.

Data Analysis
First, frequency data was generated. Next, we used logistic regressions to calculate the odds ratios (OR) with 95% confidence intervals (CI) of autism, intellectual disabilities, age, and gender in predicting (1) having a mental health condition, and (2) poor general health. We dichotomised the general health status variable to good health (very good or good health) or poor health (fair, bad, or very bad health). The gender variable was binary, the reference group was male. We conducted the analyses separately for children and young people (aged 0–24 years) and adults (aged 25+years). This was because in Scotland's Census, 2011, the prevalence of autism is higher in the children and young people than in the adults, most likely due to widening out of the diagnostic criteria and greater awareness of autism in recent decades. Hence the adults with autism are more likely to be on the more severely affected range of the autism spectrum. For the children and young people, the reference group was aged 0-15 years (childhood). The adults were grouped into 10 year age-bands, with the reference group being aged 25-34 years. We then conducted a second round of the regressions, including the interaction terms age x intellectual disabilities and age x autism. This was because the influence of age on mental health and general health is likely to differ in people with intellectual disabilities and possibly in people with autism to that seen in other people. All analyses were conducted with SPSS software version 22.

For peer review only - http://bmjopen.bmj.com/site/about/guidelines.xhtml

For peer review only

RESULTS

Scotland's Census, 2011, includes records on 5,295,403 people aged 0-75+ years, of whom 1,548,819 were children and young people, and 3,746,584 were adults aged 25 years and over. Of the children and young people, 9,396 (0.6%) reported having intellectual disabilities and 25,063 (1.6%) reported having autism. Of the adults aged 25 years and over, 16,953 (0.5%) reported having intellectual disabilities and 6,649 (0.2%) reported having autism.

9.5% of children and young people with intellectual disabilities but no autism, and 22.5% of adults with intellectual disabilities but no autism had a mental health condition. 7.5% of children and young people with autism but no intellectual disabilities, and 28% of adults with autism but no intellectual disabilities had a mental health condition. 1% of the children and young people with neither condition, and 5.7% of the adults with neither condition had a mental health condition.

Table 1 presents the OR (95% CI) of intellectual disabilities, autism, age, and gender in predicting a mental health condition in the children and young people. It presents the results of two regressions, the second one including the interaction terms. Both intellectual disabilities (OR=7.0) and autism (OR=25.1) independently increased the odds of having a mental health condition, more so for autism. Mental health conditions were also predicted by female gender (OR=1.5) and being a young person rather than a child (OR=10.5).

- Insert table 1 about here –

In adults (table 2), a similar pattern was seen with both intellectual disabilities (OR=3.5) and autism (OR=5.3) independently predicting a mental health condition, as did female gender (OR=1.3). All age groups had higher odds ratios than the 25-34 year olds of predicting having a mental health condition, except for the oldest age group, aged 65+ years who had a lower rate.

- Insert table 2 about here –

43.5% of children and young people with intellectual disabilities but no autism, and 43.1% of adults with intellectual disabilities but no autism had poor general health. 18.3% of children and young people with autism but no intellectual disabilities, and 9.6% of adults with autism but no intellectual disabilities had poor general health. 2.8% of the children and young people with neither condition, and 23.6% of the adults with neither condition had poor general health.

Table 3 presents the OR (95% CI) of intellectual disabilities, autism, age, and gender in predicting poor general health in the children and young people. It presents the results of two regressions, the second one including the interaction terms. Both intellectual disabilities (OR=18.3) and autism (OR=8.4) independently increased the odds of having poor general health, more so for intellectual disabilities. Poor general health was also predicted by female gender (OR=1.1) and being a young person rather than a child (OR=2.3).

- Insert table 3 about here –

For peer review only - http://bmjopen.bmj.com/site/about/guidelines.xhtml

In adults (table 4), a similar pattern was seen with both intellectual disabilities (OR=7.5) and autism (OR=4.5) independently predicting poor general health, as did female gender (OR=1.1). A gradient is seen, with older age groups progressively predicting having poor general health.

- Insert table 4 about here –

DISCUSSION

Principle findings and interpretation

This is the largest study to-date on this topic, comprising a whole country population. Our findings have demonstrated that both intellectual disabilities and autism are substantially associated with having a mental health condition and with poor general health, in children/young people and in adults, after the overlap between these two conditions (intellectual disabilities and autism) is accounted for. For mental health conditions, this is particularly so for autism (OR=25.1 for children/young people; OR=5.3 for adults). For poor general health, this is particularly so for intellectual disabilities (OR=18.3 for children/young people; OR=7.5 for adults). Previous literature on this is limited, and has not taken account of the overlap between autism and intellectual disabilities. It is of particular note that autism contributes substantially to poor general health and especially to having a mental health condition even after taking account of the contribution of intellectual disabilities. The mental health conditions did not include transient common mental disorders, as the question referred to mental health conditions lasting or expected to last at least 12 months, i.e. severe mental health conditions.

The extent of mental and general health inequality is greatest in children and young people with autism or with intellectual disabilities than it is for adults, though is substantial at all ages. This reflects that both mental health conditions and poor general health are much more common in adults than children and young people in the general population, whilst they are common at all ages in people with autism and in people with intellectual disabilities. Indeed, in people with intellectual disabilities, those with more severe intellectual disabilities have more comorbidity[16] and die at an earlier age[17] including in childhood. Hence, with increasing age, although acquiring age-related conditions, the population with intellectual disabilities has less disability-related comorbidity and perversely may be healthier than the younger population with intellectual disabilities.

Comparison with previous literature

No previous studies have been identified which investigated the extent to which autism and intellectual disabilities are independently associated with poor mental and general health, in children and adults. We believe this is therefore the first study to do so and subsequently we cannot compare these results.

Strengths and limitations

The main strengths of the study are the 94% whole population response, rather than biased sampling; the large population size of 5.3 million; that the conditions (intellectual disabilities, autism, mental health condition, general health) were systematically enquired about for each person; and that the phrasing of the questions underwent cognitive question testing in advance of the Census to ensure they captured the intended meaning. Consequently, we believe these results are generalisable to other high-income countries. Limitations include

For peer review only - http://bmjopen.bmj.com/site/about/guidelines.xhtml

that conditions were self/proxy reports rather than in-depth diagnostic assessments (which would not be possible on such a large scale). With regards to intellectual disabilities and autism, these conditions are typically diagnosed during infant/primary school age, if not before, as in Scotland these diagnoses attract additional educational support which is to the child's advantage; once diagnosed these are lifetime diagnoses. Consequently, there may be an undercount in the early years of childhood, whereas reporting of these conditions should be accurate in later childhood, youth, and in adults, within the diagnostic criteria prevailing at the time of diagnosis. The proportion of people in the population reported to have autism was lower after age 25, reflecting the broadening of diagnostic criteria and greater awareness of autism in recent years; hence the older people with autism might have more severe autism than the children/youth reported to have autism. We do not know the extent to which reporting of mental health conditions and general health status would reflect that found in in-depth diagnostic assessments, although subjective general health status is commonly used in population studies, and it is well established as an extremely valid measure of health; there is a strongly predictive linear gradient across subjective health status and subsequent number of medical appointments, hospital admissions, and mortality.[18-21] We do not know the proportion who self-reported or for whom the report was by another household reference person (e.g. parent); however, the latter is likely to be more common for the people with intellectual disabilities in view of their intellectual disabilities, and for the children and young people. 6% of Census entries were imputed. The Census team assumed the 2.6% who did not provide information on long-term conditions did not have any of them, but we are unable to confirm the accuracy of this assumption.

Implications

Intellectual disabilities and autism are not uncommon, and due to their associated poor mental and general health, services and supports need to be available in sufficient quantity and quality. Our findings demonstrate that this is not just related to the co-existence of these conditions, or just to having intellectual disabilities, as autism is also independently associated with substantial health inequalities across the entire life-course.

Competing interests

All authors have completed the Unified Competing Interest form (available on request from the corresponding author) and declare: no support from any organisation for the submitted work; no financial relationships with any organisations that might have an interest in the submitted work in the previous three years, no other relationships or activities that could appear to have influenced the submitted work."

Details of contributors

DK analysed the data, jointly interpreted it, and wrote the first draft of the manuscript, ER jointly interpreted the data, and contributed to the manuscript, KD jointly interpreted the data, and contributed to the manuscript, LHMc jointly interpreted the data, and contributed to the manuscript, CM jointly conceived the project, interpreted the data, and contributed to the manuscript, AH jointly interpreted the data, and contributed to the manuscript, S-AC jointly conceived the project, interpreted the data, and contributed to the manuscript. All authors approved the final version of the manuscript. S-AC is the study guarantor.

For peer review only - http://bmjopen.bmj.com/site/about/guidelines.xhtml

Statements

All authors had full access to all of the data (including statistical reports and tables) in the study and can take responsibility for the integrity of the data and the accuracy of the data analysis.

The Corresponding Author has the right to grant on behalf of all authors and does grant on behalf of all authors, a worldwide licence to the Publishers and its licensees in perpetuity, in all forms, formats and media (whether known now or created in the future), to i) publish, reproduce, distribute, display and store the Contribution, ii) translate the Contribution into other languages, create adaptations, reprints, include within collections and create summaries, extracts and/or, abstracts of the Contribution, iii) create any other derivative work(s) based on the Contribution, iv) to exploit all subsidiary rights in the Contribution, v) the inclusion of electronic links from the Contribution to third party material where-ever it may be located; and, vi) licence any third party to do any or all of the above."

Transparency declaration

The manuscript's guarantor (S-AC) affirms that the manuscript is an honest, accurate, and transparent account of the study being reported; that no important aspects of the study have been omitted; and that there were no discrepancies from the study as planned.

Data sharing

No additional data available.

Acknowledgements

We acknowledge funding from the UK Medical Research Council (grant number: MC_PC_1717).

REFERENCES

1. Cooper S-A, Smiley E, Morrison J, Allan L, Williamson A. Prevalence of and associations with mental ill-health in adults with intellectual disabilities. *Br J Psychiatry* 2007; **190**: 27-35.
2. Emerson E, Hatton C. The mental health of children and adolescents with intellectual disabilities in Britain. *Br J Psychiatry* 2007; **191**: 493–9.
3. Hughes-McCormack LA, Rydzewska E, Henderson A, Rintoul J, Macintyre C, Cooper S-A. Prevalence of mental ill-health and relationship with physical health in a whole country population of 26,349 people with intellectual disabilities, compared with the general population. *Br J Psychiatry Open* 2017; **3(5)**: http://bjpo.rcpsych.org/content/3/5/243
4. Hughes-McCormack LA, Rydzewska E, Henderson A, Rintoul J, Macintyre C, Cooper S-A. Prevalence and general health status of people with intellectual disabilities in Scotland -a total population study. *J Epidemiol Community Health* 2017; **72**: 78–85.
5. National Longitudinal Transition Study 2 (NLTS2). *Data Tables NLTS2 Waves 1 – 5; 2017*. http://www.nlts2.org/data_tables/index.html
6. Simonoff E, Pickles A, Charman T, Chandler S, Loucas T, Baird G. Psychiatric disorders in children with autism spectrum disorders: Prevalence, comorbidity and associated factors in a population-derived sample. *J Am Academy Child Adolescent Psychiatry* 2008; **47**: 921–29.

For peer review only - http://bmjopen.bmj.com/site/about/guidelines.xhtml

7. Rydzewska E, Hughes-McCormack LA, Gillberg C, Henderson A, MacIntyre C, Rintoul J, et al. Prevalence of long-term health conditions in adults with autism - observational study of a whole country population. *BMJOpen* 2018; doi:10.1136/bmjopen-2018-023945
8. Croen LA, Zerbo O, Qian Y, Massolo ML, Rich S, Sidney S, Kripke C. The health status of adults on the autism spectrum. *Autism* 2015; **19**: 814–23.
9. Rydzewska E, Hughes-McCormack LA, Gillberg C, Henderson A, MacIntyre C, Rintoul J, et al. Prevalence of sensory impairments, physical and intellectual disabilities, and mental health in children and young people with self/proxy-reported autism - observational study of a whole country population. *Autism,* 2018, (doi:10.1177/1362361318791279)
10. Fombonne E. Epidemiological Surveys of Autism and Other Pervasive Developmental Disorders: An Update. *J Autism Developmental Disorders* 2003; **33**: 365-82.
11. Ghirardi L, Brikell I, Kuja-Halkola R, Freitag CM, Franke B, Asherson P, Lichtenstein P, Larsson H. The familial co-aggregation of ASD and ADHD: a register-based cohort study, *Molecular Psychiatry* 2018; **23(2)**: 257-62.
12. Brugha TS, Spiers N, Bankart J, Cooper S-A, McManus, S, Scott F, et al. Epidemiology of autism in adults across age groups and ability levels. *Br J Psychiatry* 2016; **209**: 498-503.
13. Melville CA, Cooper S-A, Morrison J, Smiley E, Allan L , Jackson A, et al. The prevalence and incidence of mental ill-health in adults with autism and intellectual disabilities. *J Autism Developmental Disorders* 2008; **38**: 1678-88.
14. National Records of Scotland. *2011 Census Release 1C - How the 2011 Census population estimates were obtained*. Edinburgh: National Records of Scotland 2013.
15. Willis GB. *Cognitive Interviewing. A tool for improving questionnaire design.* Sage Publications. Thousand Oaks, 2005.
16. Kinnear, D, Morrison J, Allan L, Henderson A, Smiley E, Cooper. S.A. Multi-morbidity in a cohort of adults with intellectual disabilities, with and without Down syndrome. *BMJOpen* 2017; doi:10.1136/bmjopen-2017-018292
17. O'Leary L, Hughes-McCormack L, Cooper S-A. Early death and causes of death of people with intellectual disabilities: A systematic review. *J Applied Res in Intellectual Disabilities* 2017;1(3):325-42.
18. Miilunpalo S, Vuori I, Oja P, Pasanen M, Urponen H. Self-rated Health Status as a Health Measure: The Predictive Value of Self-reported Health Status on the Use of Physician Services and on Mortality in the Working-age Population. *J Clinical Epidemiol* 1997; **50(5)**: 517–28.
19. Burnstrom B, Fredlund P. Self rated health: is it as good a predictor of subsequent mortality among adults in lower as well as in higher social classes? *Jof Epidemiol Community Health* 2001; **55(11)**: 836-40.
20. Heistaro S, Jousilahti P, Lahelma E, Vartiainen E, Puska P. Self rated health and mortality: a long term prospective study in Eastern Finland. *Epidemiol Community Health* 2001; **55**: 227-32.
21. Schnittker J, Bacak V. The increasing predictive validity of self-rated health. *PLOS One* 2014; https://doi.org/10.1371/journal.pone.0084933

For peer review only - http://bmjopen.bmj.com/site/about/guidelines.xhtml

Table 1. Predictors of mental health conditions in the whole population of children and young people

Variable		Regression 1		Regression 2 (including interaction terms)	
		**Odds ratio**	**95% CI**	**Odds ratio**	**95% CI**
Age	0-15 (ref)	-		-	
	16-24	7.65	7.36-7.95	10.54	10.06-11.05
Gender	Male (ref)	-		-	
	Female	1.49	1.45-1.54	1.48	1.44-1.53
Autism	No autism (ref)	-		-	
	Autism	10.21	9.67-10.78	25.08	23.02-27.32
Intellectual disabilities	No intellectual disabilities (ref)	-		-	
	Intellectual disabilities	5.85	5.44-6.29	7.04	6.30-7.87
Age x intellectual disabilities	0-15 (ref)	-		-	
	16-24	-		0.66	0.57-0.76
Age x autism	0-15 (ref)	-		-	
	16-24	-		0.24	0.21-0.26
Constant		0.00		0.00	

For peer review only

Table 2. Predictors of mental health conditions in the whole population of adults

Variable		Regression 1		Regression 2 (including interaction terms)	
		Odds ratio	95% CI	Odds ratio	95% CI
Age	25-34 (ref)	-		-	
	35-44	1.40	1.38-1.42	1.40	1.38-1.42
	45-54	1.38	1.36-1.40	1.38	1.36-1.40
	55-64	1.08	1.06-1.09	1.07	1.05-1.08
	65+	0.92	0.90-0.93	0.91	0.90-0.92
Gender	Male (ref)	-		-	
	Female	1.25	1.24-1.26	1.25	1.24-1.26
Autism	No autism (ref)	-		-	
	Autism (ref)	5.29	5.00-5.59	5.30	4.80-5.85
Intellectual disabilities	No intellectual disabilities (ref)			-	
	Intellectual disabilities	4.42	4.26-4.59	3.50	3.20-3.84
Age x intellectual disabilities	25-34 (ref)	-		-	
	35-44	-		0.99	0.87-1.11
	45-54	-		1.24	1.10-1.39
	55-64	-		1.71	1.51-1.94
	65+	-		1.82	1.59-2.08
Age x autism	25-34 (ref)	-		-	
	35-44	-		1.03	0.89-1.19
	45-54	-		0.94	0.80-1.10
	55-64	-		1.02	0.84-1.25
	65+	-		1.18	0.97-1.44
Constant		0.46		0.46	

For peer review only - http://bmjopen.bmj.com/site/about/guidelines.xhtml

Table 3. Predictors of poor general health in the whole population of children and young people

Variable		Regression 1		Regression 2 (including interaction terms)	
		Odds ratio	95% CI	Odds ratio	95% CI
Age	0-15 (ref)	-		-	
	16-24	2.14	2.10-2.18	2.28	2.24-2.33
Gender	Male (ref)	-		-	
	Female	1.11	1.09-1.14	1.11	1.09-1.13
Autism	No autism (ref)	-		-	
	Autism	6.70	6.46-6.95	8.40	8.02-8.80
Intellectual disabilities	No intellectual disabilities (ref)	-		-	
	Intellectual disabilities	14.05	13.39-14.73	18.34	17.17-19.58
Age x intellectual disabilities	0-15 (ref)	-		-	
	16-24	-		0.57	0.52-0.63
Age x autism	0-15 (ref)	-		-	
	16-24	-		0.54	0.50-0.58
Constant		0.02		0.02	

For peer review only

For peer review only - http://bmjopen.bmj.com/site/about/guidelines.xhtml

Table 4. Predictors of poor general health in the whole population of adults

Variable		Regression 1		Regression 2 (including interaction terms)	
		Odds ratio	95% CI	Odds ratio	95% CI
Age	25-34 (ref)	-		-	
	35-44	1.78	1.76-1.80	1.79	1.77-1.81
	45-54	2.86	2.83-2.89	2.90	2.87-2.93
	55-64	4.81	4.76-4.86	4.88	4.82-4.93
	65+	10.25	10.15-10.36	10.39	10.29-10.50
Gender	Male (ref)	-		-	
	Female	1.05	1.05-1.06	1.05	1.05-1.06
Autism	No autism (ref)	-		-	
	Autism (ref)	3.39	3.21-3.58	4.46	4.06-4.89
Intellectual disabilities	No intellectual disabilities (ref)			-	
	Intellectual disabilities	4.39	4.25-4.53	7.54	7.02-8.10
Age x intellectual disabilities	25-34 (ref)	-		-	
	35-44	-		0.72	0.65-0.79
	45-54	-		0.60	0.54-0.65
	55-64	-		0.45	0.40-0.50
	65+	-		0.24	0.21-0.26
Age x autism	25-34 (ref)	-		-	
	35-44	-		0.83	0.72-0.96
	45-54	-		0.59	0.50-0.68
	55-64	-		0.49	0.41-0.59
	65+	-		0.44	0.36-0.53
Constant		0.08		0.08	

For peer review only

For peer review only - http://bmjopen.bmj.com/site/about/guidelines.xhtml

BMJ Open

The relative influence of intellectual disabilities and autism on mental and general health in Scotland. A cross-sectional study of a whole country of 5.3 million children and adults

Journal:	*BMJ Open*
Manuscript ID	bmjopen-2019-029040.R1
Article Type:	Research
Date Submitted by the Author:	06-Jun-2019

Complete List of Authors:	Kinnear, Deborah; University of Glasgow, Institute of Health and Wellbeing<br>Rydzewska, Ewelina; University of Glasgow, Institute of Health and Wellbeing<br>Dunn, Kirsty; University of Glasgow, Institute of Health and Wellbeing<br>Hughes-McCormack, Laura; University of Glasgow, Institute of Health and Wellbeing<br>Melville, Craig; University of Glasgow<br>Henderson, Angela; University of Glasgow, Institute of Health and Wellbeing<br>Cooper, Sally-Ann; Glasgow University , Institute of Health and Wellbeing

**Primary Subject Heading**:	Epidemiology
Secondary Subject Heading:	Health informatics
Keywords:	intellectual disabilities, autism, MENTAL HEALTH, general health

SCHOLARONE™ Manuscripts

The relative influence of intellectual disabilities and autism on mental and general health in Scotland. A cross-sectional study of a whole country of 5.3 million children and adults

***Deborah Kinnear[1] PhD**
Ewelina Rydzewska[1] PhD
Kirsty Dunn[1] MA
Laura Hughes-McCormack[1] MA
Craig Melville[1], MD FRCPsych
Angela Henderson[1] BSc
Sally-Ann Cooper[1] MD FRCPsych

*Correspondence: Institute of Health and Wellbeing, University of Glasgow, Mental Health and Wellbeing Group, 1st Floor Administrative Building, Gartnavel Royal Hospital, 1055 Great Western Road, Glasgow G12 0XH, UK
Deborah.Kinnear@glasgow.ac.uk
[1]Mental Health and Wellbeing Research Group, Institute of Health and Wellbeing, University of Glasgow, Glasgow, UK

Word Count: 2,988

ABSTRACT

Objectives: To determine the relative extent that autism and intellectual disabilities are independently associated with poor mental and general health, in children and adults.
Design: Cross-sectional study. For Scotland's population, logistic regressions investigated odds of intellectual disabilities and autism predicting mental health conditions, and poor general health, adjusted for age and gender.
Participants: 1,548,819 children/youth aged 0-24y, and 3,746,584 adults aged 25y+, of whom 9,396/1,548,819 children/youth had intellectual disabilities (0.6%), 25,063/1,548,819 children/youth had autism (1.6%); and 16,953/3,746,584 adults had intellectual disabilities (0.5%), 6,649/3,746,584 adults had autism (0.2%). These figures are based on self-report.
Main outcome measures: Self-reported general health status and mental health
Results: In children/youth, intellectual disabilities (OR=7.04; CI 6.30, 7.87) and autism (OR=25.08, CI 23.08, 27.32) both independently predicted mental health conditions. In adults, intellectual disabilities (OR=3.50; CI 3.20, 3.84), and autism (OR=5.30, CI 4.80, 5.85) both independently predicted mental health conditions. In children/youth, intellectual disabilities (OR=18.34, CI 17.17, 19.58) and autism (OR=8.40, CI 8.02, 8.80) both independently predicted poor general health. In adults, intellectual disabilities (OR=7.54, CI 7.02, 8.10), and autism (OR=4.46, CI 4.06, 4.89) both independently predicted poor general health.
Conclusions: Both intellectual disabilities and autism independently predict poor health, intellectual disabilities more so for general health, and autism more so for mental health. Intellectual disabilities and autism are not uncommon, and due to their associated poor health, sufficient services/supports are needed. This is not just due to co-existence of these conditions, or just to having intellectual disabilities, as the population with autism is independently associated with substantial health inequalities compared with the general population, across the entire life-course

For peer review only - http://bmjopen.bmj.com/site/about/guidelines.xhtml

Key words: intellectual disabilities, autism, mental health, general health

Strengths and limitations of this study

- This large study is the first study to have reported on the extent to which autism and intellectual disabilities are independently associated with poor mental and general health, in children and adults.
- The study comprises a whole country population, with high participation rate (94%), and the conditions were systematically enquired about on everyone.
- A limitation is that conditions were self/proxy reports rather than in-depth diagnostic assessments.

For peer review only

INTRODUCTION

Both intellectual disabilities and autism occur not uncommonly in children and adults, and can co-occur. Children and adults with intellectual disabilities have notably poorer mental and general health than other people.[1-4] This has also been reported for autistic children and adults,[5-9] although the quantity of research is limited, particularly with regards to adults. The extent of co-occurrence of intellectual disabilities in autistic people used to be considered to be as high as 50-70%,[10] although more recent reports suggest it may be lower, though still considerable, at about 20%.[9,11] This may in part relate to the broadening of criteria for the autism spectrum to include “milder” autism and greater awareness about autism in children and young people in recent years, as it is well established that autism prevalence is higher in people with more severe intellectual disabilities and vice-versa.[12] Autism is generally considered to be associated with poor mental health. However, the largest study to examine this in adults in a general community population found no difference in rates of mental ill-health in adults with co-occurring autism and intellectual disabilities, compared with age-gender-Down syndrome-level of ability-matched adults with intellectual disabilities but no autism.[13] As intellectual disabilities and autism have tended to be studied separately, the relative extent to which being autistic, or having intellectual disabilities, accounts for their poor population health is not clear. This is important to understand, given the frequent co-occurrence of these conditions, and is important to understand in both child and adult populations, given the more recent change in co-occurrence due to higher frequency of diagnosis of autism.

The aim of this paper is to study the extent to which autism and intellectual disabilities are independently associated with poor mental and general health, in children and adults.

METHOD

STROBE guidelines

The STrengthening the Reporting of Observational studies in Epidemiology (STROBE) checklist for cross-sectional studies was adhered to.

Approval

For peer review only - http://bmjopen.bmj.com/site/about/guidelines.xhtml

Approval was gained from the Scottish Government for secondary analysis of the Scotland Census, 2011 data. Access to a subset of data was provided.

Census process and variables

Scotland has performed a national Census every 10 years since 1841, the most recent being Scotland's Census, 2011. Information was collected on every resident in Scotland on the Census date, 27.3.11. This included people in private households and also people in community residences (such as care homes, prisons, student halls of residence). In private households (typically family households), one person was responsible for completing the Census details for all the household's residents; for communal establishments the manager was responsible for providing the information. It is a legal requirement in the UK to complete the Census. Failure to provide information, or for providing false information attracted a fine of up to £1,000. Non-responses were followed up by the Census team and help provided. These factors accounted for the high response rate; Scotland's Census 2011 achieved a 94% response rate.[14] The Census team adjusted for the 6% non-response rate using a Census Coverage Survey to estimate numbers and characteristics. The Census Coverage Survey included around 40,000 households; the records from it were matched with Census records, with all individuals deterministically matched to check for duplicates. Individuals estimated to be missing from the Census were then imputed, using a subset of characteristics from real individuals, including health information. This Edit and Imputation Methodology was adapted from the Office for National Statistics rigorous and systematic guidelines, available at:
http://webarchive.nationalarchives.gov.uk/20160108193745/http://www.ons.gov.uk/ons/guide-method/method-quality/survey-methodology-bulletin/smb-69/index.html
and further details on the Census population estimates are available at:
http://www.scotlandscensus.gov.uk/documents/censusresults/release1b/rel1bmethodology.pdf
Full details of the methodology and other background information on Scotland's Census, 2011 are available at:
http://www.scotlandscensus.gov.uk/supporting-information.

The Census included questions on demography, long-term conditions, and on general health.

The question on long-term conditions enquired:
"Do you have any of the following conditions which have lasted, or are expected to last, at least 12 months? Tick all that apply:

- Deafness or partial hearing loss
- Blindness or partial sight loss
- Learning disability (for example, Down's Syndrome)
- Learning difficulty (for example, dyslexia)
- Developmental disorder (for example, Autistic Spectrum Disorder or Asperger's Syndrome)
- Physical disability
- Mental health condition
- Long-term illness, disease or condition
- Other condition, please write in
  - free text space was then provided for conditions to be listed

or

- No condition"

For peer review only - http://bmjopen.bmj.com/site/about/guidelines.xhtml

The question on general health enquired:
"How is your health in general?
- Very good
- Good
- Fair
- Bad
- Very bad"

The terminology used in both these questions was specifically investigated prior to implementation of data collection. The General Register Office for Scotland commissioned Ipsos MORI Scotland to undertake cognitive question testing, to determine whether the questions were answered accurately and willingly by respondents, and what changes if any might be required to improve data quality and/or the acceptability of the response options. Cognitive interviewing is a widely used approach to critically evaluate survey questionnaires.[15] It tests the way respondents understand, mentally process, and respond to survey materials. It enables researchers to modify survey material to enhance clarity. Retrospective probing was deemed to be the most appropriate of the different techniques available. It involved the interviewer presenting the question, the respondent answering it, and the interviewer then probing for specific information relevant to the question or to the specific answer given (e.g. What does this question mean in your own words?)." This research was undertaken with 102 participants with a mix of gender and age, both with and without the health conditions and disabilities (including people with more than one of the conditions). This included people with autism, intellectual disabilities, dyslexia, dyspraxia, speech impairment, mental health conditions (both milder and more serious), and other long-term conditions. The results found that the question on general health status functioned well and did not need amendment, as did the questions on long-term conditions, including intellectual disabilities and mental health condition, whilst the question on autism was redesigned to that listed above in order to more accurately capture the data specifically on autism. Additionally, the response "no" was amended to "no condition". The other questions did not require any modification. Further information can be found at:

http://www.scotlandscensus.gov.uk/documents/research/2011-census-health-disability-questions.pdf
http://www.scotlandscensus.gov.uk/documents/legislation/changes-to-gov-statement-report.pdf

In Scotland, the term "learning disability" is synonymous with the international term "intellectual disabilities".[16,17]

For 2.6% of the Census returns, information on long-term conditions was not completed. The Census team assumed the most plausible explanation was that the person had no long-term condition but did not see the 'No condition' check box at the end of the question. They thus recorded them to have none of the long-term conditions.

Data Analysis
First, frequency data was generated. Next, we used logistic regressions to calculate the odds ratios (OR) with 95% confidence intervals (CI) of autism, intellectual disabilities, age, and gender in predicting (1) having a mental health condition, and (2) poor general health. We

For peer review only - http://bmjopen.bmj.com/site/about/guidelines.xhtml

dichotomised the general health status variable to good health (very good or good health) or poor health (fair, bad, or very bad health). The gender variable was binary, the reference group was male. We conducted the analyses separately for children and young people (aged 0–24 years) and adults (aged 25+years). This was because in Scotland's Census, 2011, the prevalence of autism is higher in the children and young people than in the adults, most likely due to widening out of the diagnostic criteria and greater awareness of autism in recent decades. Hence the adults with autism are more likely to be on the more severely affected range of the autism spectrum. For the children and young people, the reference group was aged 0-15 years (childhood), given the physiological changes and changing life experiences that occur in adolescence/transition compared with younger children, which may have a bearing on general and mental health,. The adults were grouped into 10 year age-bands, with the reference group being aged 25-34 years. We then conducted a second round of the regressions, including the interaction terms age x intellectual disabilities and age x autism. This was because the influence of age on mental health and general health is likely to differ in people with intellectual disabilities and possibly in people with autism to that seen in other people. All analyses were conducted with SPSS software version 22.

Patient and Public Involvement
The question on intellectual disabilities and autism was included in Scotland's Census, 2011 at the behest of third sector organisations for people with intellectual disabilities and autism. This study was undertaken by the Scottish Learning Disabilities Observatory, which has a specific remit for people with intellectual disabilities and autism; its steering group includes partners from the third sector organisations. Results from this study will be disseminated for people with intellectual disabilities and autism in easy-read version via the Scottish Learning Disabilities Observatory website and newsletters.

RESULTS

Scotland's Census, 2011, includes records on 5,295,403 people aged 0-75+ years, of whom 1,548,819 (29.2%) were children and young people, and 3,746,584 (70.8%) were adults aged 25 years and over. Of the children and young people, 9,396 (0.6%) reported having intellectual disabilities and 25,063 (1.6%) reported having autism. Of the adults aged 25 years and over, 16,953 (0.5%) reported having intellectual disabilities and 6,649 (0.2%) reported having autism. Of the children and young people with intellectual disabilities, 3,756/9,396 (40.0%) additionally had autism, and of the adults aged 25 years and over with intellectual disabilities, 1953/16,953 (11.5%) additionally had autism. Of the children and young people with autism, 3,756/25,063 (15.0%) additionally had intellectual disabilities, and of the adults aged 25 years and over with autism, 1,953/6,649 (29.4%) additionally had intellectual disabilities.

538/5,640 (9.5%) of the children and young people with intellectual disabilities but no autism had a mental health condition, and 3,383/15,000 (22.6%) of the adults with intellectual disabilities but no autism had a mental health condition. 1,601/21,307 (7.5%) of the children and young people with autism but no intellectual disabilities had a mental health condition, and 1,314/4,696 (28.0%) of adults with autism but no intellectual disabilities had a mental health condition. 15,829/1,518,116 (1.0%) of the children and young people with neither condition had a mental health condition, and 208,493/3,724,935 (5.6%) of the adults with neither condition had a mental health condition.

For peer review only - http://bmjopen.bmj.com/site/about/guidelines.xhtml

Table 1 presents the OR (95% CI) of intellectual disabilities, autism, age, and gender in predicting a mental health condition in the children and young people. It presents the results of two regressions, the second one including the interaction terms. Both intellectual disabilities OR=7.0 (6.3-7.9) and autism OR=25.1 (23.0-27.3) independently increased the odds of having a mental health condition, more so for autism. Mental health conditions were also predicted by female gender OR=1.5 (1.4-1.5) and being a young person rather than a child OR=10.5 (10.1-11.0).

- Insert table 1 about here –

In adults (table 2), a similar pattern was seen with both intellectual disabilities OR=3.5 (3.2-3.8) and autism OR=5.3 (4.8-5.9) independently predicting a mental health condition, as did female gender OR=1.3 (1.2-1.3). All age groups had higher odds ratios than the 25-34 year olds of predicting having a mental health condition, except for the oldest age group, aged 65+ years who had a lower rate.

- Insert table 2 about here –

2,453/5,640 (43.5%) of children and young people with intellectual disabilities but no autism, and 7,834/15,000 (52.2%) of adults with intellectual disabilities but no autism had poor general health. 3,898/21,307 (18.3%) of children and young people with autism but no intellectual disabilities, and 2,134/4,696 (45.4%) of adults with autism but no intellectual disabilities had poor general health. 42,713/1,518,116 (2.8%) of the children and young people with neither condition, and 880,044/3,724,935 (23.6%) of the adults with neither condition had poor general health.

Table 3 presents the OR (95% CI) of intellectual disabilities, autism, age, and gender in predicting poor general health in the children and young people. It presents the results of two regressions, the second one including the interaction terms. Both intellectual disabilities OR=18.3 (17.2-19.6) and autism OR=8.4 (8.0-8.8) independently increased the odds of having poor general health, more so for intellectual disabilities. Poor general health was also predicted by female gender OR=1.1 (1.1-1.1) and being a young person rather than a child OR=2.3 (2.2-2.3).

- Insert table 3 about here –

In adults (table 4), a similar pattern was seen with both intellectual disabilities OR=7.5 (7.0-8.1) and autism OR=4.5 (4.1-4.9) independently predicting poor general health, as did female gender OR=1.1 (1.1-1.1). A gradient is seen, with older age groups progressively predicting having poor general health.

- Insert table 4 about here –

DISCUSSION

Principle findings and interpretation

This is the largest study to-date on this topic, comprising a whole country population. Our findings have demonstrated that both intellectual disabilities and autism are associated with having a mental health condition and with poor general health. This is so in both

For peer review only - http://bmjopen.bmj.com/site/about/guidelines.xhtml

children/young people and in adults, after the overlap between these two conditions (intellectual disabilities and autism) is accounted for. For mental health conditions, this is particularly so for autism (OR=25.1 (23.0-27.3) for children/young people; OR=5.3 (4.8-5.9) for adults). For poor general health, this is particularly so for intellectual disabilities (OR=18.3 (17.2-19.6) for children/young people; OR=7.5 (7.0-8.1) for adults). Previous literature on this is limited, and has not taken account of the overlap between autism and intellectual disabilities. It is of particular note that autism contributes to poor general health and especially to having a mental health condition even after taking account of the contribution of intellectual disabilities. The mental health conditions did not include transient common mental disorders, as the question referred to mental health conditions lasting or expected to last at least 12 months, i.e. severe mental health conditions.

The extent of mental and general health inequality experienced by the population with intellectual disabilities and the population with autism, in comparison with the general population, is greatest in children/young people than it is for adults, though is substantial at all ages. This reflects that both mental health conditions and poor general health are much more common in adults than children and young people in the general population, whilst they are common at all ages in people with autism and in people with intellectual disabilities. Indeed, in people with intellectual disabilities, those with more severe intellectual disabilities have more comorbidity[18] and die at an earlier age[19] including in childhood. Hence, with increasing age, although acquiring age-related conditions, the population with intellectual disabilities has less disability-related comorbidity and perversely may be healthier than the younger population with intellectual disabilities.

The aetiology of mental and general ill-health in people with intellectual disabilities or people with autism includes genetic predisposition[18]; indeed the term ESSENCE (Early Symptomatic Syndromes Eliciting Neurodevelopmental Clinical Examination) has been coined for the association of problems in one or more of ten health domains in young children.[19] It is clear though that aetiology is multifactorial, and social and environmental factors such as life events, which occur more commonly in people with intellectual disabilities, have been shown to precede onset of mental health conditions in adults with intellectual disabilities.[20] Therefore, underpinning mechanisms appear to include the interplay between genes, environment, and lifestyle[,21] including differences in health related behaviours, such as diet and exercise,[22] and inequalities in access to services.[23] This is important, as understanding these factors provides pathways to the development of interventions to improve health. Examples tailored to these populations, in addition to drug treatments, include interventions developed to address lifestyle,[24] general health,[25] and psychological interventions for mental health conditions.[26]

Comparison with previous literature

No previous studies have been identified which investigated the extent to which autism and intellectual disabilities are independently associated with poor mental and general health, in children and adults. We believe this is therefore the first study to do so and subsequently we cannot compare these results.

Strengths and limitations

The main strengths of the study are the 94% whole population response, rather than biased sampling; the large population size of 5.3 million; that the conditions (intellectual disabilities, autism, mental health condition, general health) were systematically enquired about for each

For peer review only - http://bmjopen.bmj.com/site/about/guidelines.xhtml

person; and that the phrasing of the questions underwent cognitive question testing in advance of the Census to ensure they captured the intended meaning. Consequently, we believe these results are generalisable to other high-income countries.

Limitations include the use of the term of 'developmental disorders' in the Census. However, the Census form prompted responses only for autistic spectrum disorder or Asperger's syndrome. Furthermore, the developmental disorders category was distinguished from intellectual disabilities, learning difficulties, and mental health conditions, which are important distinctions. Hence, we consider that respondents will have replied accordingly, i.e. responded regarding autism. However, we have no means to check this. In addition, conditions were self/proxy reports rather than in-depth diagnostic assessments (which would not be possible on such a large scale). Respondents reported whether or not each person was known to have autism and/or intellectual disabilities, rather than each person having an assessment, so some reporting error is possible. However, intellectual disabilities and autism are conditions that are typically diagnosed during infant/primary school age, if not before. In Scotland these diagnoses attract additional educational support which is to the child's advantage; once diagnosed these are lifetime diagnoses. Consequently, there may be an undercount in the early years of childhood, whereas reporting of these conditions should be accurate in later childhood, youth, and in adults, within the diagnostic criteria prevailing at the time of diagnosis. The proportion of people in the population reported to have autism was lower after age 25. This reflects the broadening of diagnostic criteria and greater awareness of autism in recent years; hence the older people with autism might have more severe autism than the children/youth reported to have autism. The children/youth with autism are likely to include some who function well. We do not know the extent to which reporting of mental health conditions and general health status would reflect that found in in-depth diagnostic assessments, although subjective general health status is commonly used in population studies, and it is well established as an extremely valid measure of health. There is a strongly predictive linear gradient across subjective health status and subsequent number of medical appointments, hospital admissions, and mortality.[27-29] We do not know the proportion who self-reported or for whom the report was by another household reference person (e.g. parent). However, the latter is likely to be more common for the people with intellectual disabilities in view of their intellectual disabilities, and for the children and young people. 6% of Census entries were imputed. The Census team assumed the 2.6% who did not provide information on long-term conditions did not have any of them, but we are unable to confirm the accuracy of this assumption.

Future research investigating narrower age bands of children/youth may be useful, and next steps must importantly include study of the aetiology of poor health in these populations, to inform the development of further effective interventions.

Implications

Intellectual disabilities and autism are not uncommon, and due to their associated poor mental and general health, services and supports need to be available in sufficient quantity and quality. Our findings demonstrate that this is not just related to the co-existence of these conditions, or just to having intellectual disabilities, as the population with autism is also independently associated with substantial health inequalities compared with the general population, across the entire life-course.

Competing interests

For peer review only - http://bmjopen.bmj.com/site/about/guidelines.xhtml

All authors have completed the Unified Competing Interest form (available on request from the corresponding author) and declare: no support from any organisation for the submitted work; no financial relationships with any organisations that might have an interest in the submitted work in the previous three years, no other relationships or activities that could appear to have influenced the submitted work."

Details of contributors

DK analysed the data, jointly interpreted it, and wrote the first draft of the manuscript, ER jointly interpreted the data, and contributed to the manuscript, KD jointly interpreted the data, and contributed to the manuscript, LHMc jointly interpreted the data, and contributed to the manuscript, CM jointly conceived the project, interpreted the data, and contributed to the manuscript, AH jointly interpreted the data, and contributed to the manuscript, S-AC jointly conceived the project, interpreted the data, and contributed to the manuscript. All authors approved the final version of the manuscript. S-AC is the study guarantor.

Funding

This study was funded by the Medical Research Council (grant reference MC_PC_17217) and the Scottish Government via the xxx. The funders had no role in the study design, collection, analyses and interpretation of data, in writing the report, nor in the decision to submit the article for publication.

Statements

All authors had full access to all of the data (including statistical reports and tables) in the study and can take responsibility for the integrity of the data and the accuracy of the data analysis.

The Corresponding Author has the right to grant on behalf of all authors and does grant on behalf of all authors, a worldwide license to the Publishers and its licensees in perpetuity, in all forms, formats and media (whether known now or created in the future), to i) publish, reproduce, distribute, display and store the Contribution, ii) translate the Contribution into other languages, create adaptations, reprints, include within collections and create summaries, extracts and/or, abstracts of the Contribution, iii) create any other derivative work(s) based on the Contribution, iv) to exploit all subsidiary rights in the Contribution, v) the inclusion of electronic links from the Contribution to third party material where-ever it may be located; and, vi) license any third party to do any or all of the above."

Transparency declaration

The manuscript's guarantor (S-AC) affirms that the manuscript is an honest, accurate, and transparent account of the study being reported; that no important aspects of the study have been omitted; and that there were no discrepancies from the study as planned.

Data sharing

No additional data available.

Acknowledgements

We acknowledge funding from the UK Medical Research Council (grant number: MC_PC_1717).

For peer review only - http://bmjopen.bmj.com/site/about/guidelines.xhtml

REFERENCES

1. Cooper S-A, Smiley E, Morrison J, Allan L, Williamson A. Prevalence of and associations with mental ill-health in adults with intellectual disabilities. *Br J Psychiatry* 2007; 190: 27-35.
2. Emerson E, Hatton C. The mental health of children and adolescents with intellectual disabilities in Britain. *Br J Psychiatry* 2007; 191: 493–9.
3. Hughes-McCormack LA, Rydzewska E, Henderson A, Rintoul J, Macintyre C, Cooper S-A. Prevalence of mental ill-health and relationship with physical health in a whole country population of 26,349 people with intellectual disabilities, compared with the general population. *Br J Psychiatry Open* 2017; 3(5): http://bjpo.rcpsych.org/content/3/5/243
4. Hughes-McCormack LA, Rydzewska E, Henderson A, Rintoul J, Macintyre C, Cooper S-A. Prevalence and general health status of people with intellectual disabilities in Scotland -a total population study. *J Epidemiol Community Health* 2017; 72: 78–85.
5. National Longitudinal Transition Study 2 (NLTS2). *Data Tables NLTS2 Waves 1 – 5; 2017*. http://www.nlts2.org/data_tables/index.html
6. Simonoff E, Pickles A, Charman T, Chandler S, Loucas T, Baird G. Psychiatric disorders in children with autism spectrum disorders: Prevalence, comorbidity and associated factors in a population-derived sample. *J Am Academy Child Adolescent Psychiatry* 2008; 47: 921–29.
7. Rydzewska E, Hughes-McCormack LA, Gillberg C, Henderson A, MacIntyre C, Rintoul J, et al. Prevalence of long-term health conditions in adults with autism - observational study of a whole country population. *BMJOpen* 2018; doi:10.1136/bmjopen-2018-023945
8. Croen LA, Zerbo O, Qian Y, Massolo ML, Rich S, Sidney S, Kripke C. The health status of adults on the autism spectrum. *Autism* 2015; 19: 814–23.
9. Rydzewska E, Hughes-McCormack LA, Gillberg C, Henderson A, MacIntyre C, Rintoul J, et al. Prevalence of sensory impairments, physical and intellectual disabilities, and mental health in children and young people with self/proxy-reported autism - observational study of a whole country population. *Autism,* 2018, (doi:10.1177/1362361318791279)
10. Fombonne E. Epidemiological Surveys of Autism and Other Pervasive Developmental Disorders: An Update. *J Autism Developmental Disorders* 2003; 33: 365-82.
11. Ghirardi L, Brikell I, Kuja-Halkola R, Freitag CM, Franke B, Asherson P, Lichtenstein P, Larsson H. The familial co-aggregation of ASD and ADHD: a register-based cohort study, *Molecular Psychiatry* 2018; 23(2): 257-62.
12. Brugha TS, Spiers N, Bankart J, Cooper S-A, McManus, S, Scott F, et al. Epidemiology of autism in adults across age groups and ability levels. *Br J Psychiatry* 2016; 209: 498-503.
13. Melville CA, Cooper S-A, Morrison J, Smiley E, Allan L , Jackson A, et al. The prevalence and incidence of mental ill-health in adults with autism and intellectual disabilities. *J Autism Developmental Disorders* 2008; 38: 1678-88.
14. National Records of Scotland. *2011 Census Release 1C - How the 2011 Census population estimates were obtained*. Edinburgh: National Records of Scotland 2013.
15. Willis GB. *Cognitive Interviewing. A tool for improving questionnaire design.* Sage Publications. Thousand Oaks, 2005.
16. Scottish Government. The Keys to Life. Improving quality of life for people with learning disabilities. Edinburgh: Scottish Government 2013.

For peer review only - http://bmjopen.bmj.com/site/about/guidelines.xhtml

17. Truesdale M, Brown M. People with learning disabilities in Scotland: 2017 Health Needs Assessment Report. Edinburgh: NHS Health Scotland 2017.
18. Moreno-De-Luca A, Myers SM, Challman TD, Moreno-De-Luca D, Evans DW, Ledbetter DH. Developmental brain dysfunction: revival and expansion of old concepts based on new genetic evidence. Lancet Neurol 2013; 12: 406–14.
19. Gillberg C. The ESSENCE in child psychiatry: Early Symptomatic Syndromes Eliciting Neurodevelopmental Clinical Examinations. Res Dev Disabil. 2010; 31(6): 1543–51.
20. Smiley E, Cooper S-A, Finlayson J, Jackson A, Allan L, Mantry D, McGrother C, McConnachie A, Morrison J. The incidence, and predictors of mental ill-health in adults with intellectual disabilities. Prospective study. *British Journal of Psychiatry* 2007; 191, 313-319.
21. Evans GW & Kim P. Early childhood poverty and adult chronic physiological stress: The mediating role of childhood cumulative risk exposure. Psychol Sci 2012; 23(9): 979-83.
22. Borremans E, Rintala P, McCubbin JA. Physical fitness and physical activity in adolescents with asperger syndrome: a comparative study. Adapt Phys Activ Q 2010; 27(4): 308-20.
23. Cooper S-A, Hughes-McCormack L, Greenlaw N, McConnachie A, Allan L, Baltzer M, McArthur M, Henderson A, Melville C, Morrison J. Management and prevalence of long-term conditions in primary health care for adults with intellectual disabilities compared with the general population: a population-based cohort study. Journal of Applied Research in Intellectual Disabilities, 2017; 31 (S1): 68–81.
24. Willems M, Waninge A, Hilgenkamp TIM, van Empelen P, Krijnen WP, van der Schans CP and Melville CA. Effects of lifestyle change interventions for people with intellectual disabilities: Systematic review and meta-analysis of randomized controlled trials. Journal of Applied Research in Intellectual Disabilities 2018; 31(6): 949-961.
25. Cooper S-A, Morrison J, Allan L, McConnachie A, Melville C, Baltzer M, McArthur L, Lammie C, Greenlaw N, Grieves E, Fenwick E. Practice nurse health checks for adults with intellectual disabilities: a cluster design randomised controlled trial. Lancet Psychiatry 2014; 1: 511-521.
26. Jahoda, A, Hastings R, Hatton C, Cooper S-A, Dagnan D, Zhang R, McConnachie A, McMeekin N, Appleton K, Jones R, Scott K, Fulton L, Knight R, Knowles D, Williams C, Briggs A, MacMahon K, Lynn H, Smith I, Thomas G, Melville C. A randomised controlled trial comparing a behavioural activation treatment for depression in adults with intellectual disabilities with guided self-help. The Lancet Psychiatry 2017; 4: 909-919.
27. Harris, L., Mcgarty, A. M. , Hilgenkamp, T., Mitchell, F. and Melville, C. A. (2018) Correlates of objectively measured sedentary time in adults with intellectual disabilities. Preventive Medicine Reports, 9, pp. 12-17.
28. Kinnear, D, Morrison J, Allan L, Henderson A, Smiley E, Cooper. S.A. Multi-morbidity in a cohort of adults with intellectual disabilities, with and without Down syndrome. *BMJOpen* 2017; doi:10.1136/bmjopen-2017-018292
29. O'Leary L, Hughes-McCormack L, Cooper S-A. Early death and causes of death of people with intellectual disabilities: A systematic review. *J Applied Res in Intellectual Disabilities* 2017;1(3):325-42.

For peer review only - http://bmjopen.bmj.com/site/about/guidelines.xhtml

Table 1. Predictors of mental health conditions in the whole population of children and young people

Variable		Regression 1		Regression 2 (including interaction terms)	
		Odds ratio	95% CI	Odds ratio	95% CI
Age	0-15 (ref)	-		-	
	16-24	7.65	7.36-7.95	10.54	10.06-11.05
Gender	Male (ref)	-		-	
	Female	1.49	1.45-1.54	1.48	1.44-1.53
Autism	No autism (ref)	-		-	
	Autism	10.21	9.67-10.78	25.08	23.02-27.32
Intellectual disabilities	No intellectual disabilities (ref)	-		-	
	Intellectual disabilities	5.85	5.44-6.29	7.04	6.30-7.87
Age x intellectual disabilities	0-15 (ref)	-		-	
	16-24	-		0.66	0.57-0.76
Age x autism	0-15 (ref)	-		-	
	16-24	-		0.24	0.21-0.26
Constant		0.00		0.00	

For peer review only

Table 2. Predictors of mental health conditions in the whole population of adults

Variable		Regression 1		Regression 2 (including interaction terms)	
		Odds ratio	95% CI	Odds ratio	95% CI
Age	25-34 (ref)	-		-	
	35-44	1.40	1.38-1.42	1.40	1.38-1.42
	45-54	1.38	1.36-1.40	1.38	1.36-1.40
	55-64	1.08	1.06-1.09	1.07	1.05-1.08
	65+	0.92	0.90-0.93	0.91	0.90-0.92
Gender	Male (ref)	-		-	
	Female	1.25	1.24-1.26	1.25	1.24-1.26
Autism	No autism (ref)	-		-	
	Autism (ref)	5.29	5.00-5.59	5.30	4.80-5.85
Intellectual disabilities	No intellectual disabilities (ref)			-	
	Intellectual disabilities	4.42	4.26-4.59	3.50	3.20-3.84
Age x intellectual disabilities	25-34 (ref)	-		-	
	35-44	-		0.99	0.87-1.11
	45-54	-		1.24	1.10-1.39
	55-64	-		1.71	1.51-1.94
	65+	-		1.82	1.59-2.08
Age x autism	25-34 (ref)	-		-	
	35-44	-		1.03	0.89-1.19
	45-54	-		0.94	0.80-1.10
	55-64	-		1.02	0.84-1.25
	65+	-		1.18	0.97-1.44
Constant		0.46		0.46	

For peer review only - http://bmjopen.bmj.com/site/about/guidelines.xhtml

Table 3. Predictors of poor general health in the whole population of children and young people

**Variable**		**Regression 1**		**Regression 2 (including interaction terms)**	
		**Odds ratio**	**95% CI**	**Odds ratio**	**95% CI**
Age	0-15 (ref)	-		-	
	16-24	2.14	2.10-2.18	2.28	2.24-2.33
Gender	Male (ref)	-		-	
	Female	1.11	1.09-1.14	1.11	1.09-1.13
Autism	No autism (ref)	-		-	
	Autism	6.70	6.46-6.95	8.40	8.02-8.80
Intellectual disabilities	No intellectual disabilities (ref)	-		-	
	Intellectual disabilities	14.05	13.39-14.73	18.34	17.17-19.58
Age x intellectual disabilities	0-15 (ref)	-		-	
	16-24	-		0.57	0.52-0.63
Age x autism	0-15 (ref)	-		-	
	16-24	-		0.54	0.50-0.58
Constant		0.02		0.02	

For peer review only

Table 4. Predictors of poor general health in the whole population of adults

Variable		Regression 1		Regression 2 (including interaction terms)	
		Odds ratio	95% CI	Odds ratio	95% CI
Age	25-34 (ref)	-		-	
	35-44	1.78	1.76-1.80	1.79	1.77-1.81
	45-54	2.86	2.83-2.89	2.90	2.87-2.93
	55-64	4.81	4.76-4.86	4.88	4.82-4.93
	65+	10.25	10.15-10.36	10.39	10.29-10.50
Gender	Male (ref)	-		-	
	Female	1.05	1.05-1.06	1.05	1.05-1.06
Autism	No autism (ref)	-		-	
	Autism (ref)	3.39	3.21-3.58	4.46	4.06-4.89
Intellectual disabilities	No intellectual disabilities (ref)			-	
	Intellectual disabilities	4.39	4.25-4.53	7.54	7.02-8.10
Age x intellectual disabilities	25-34 (ref)	-		-	
	35-44	-		0.72	0.65-0.79
	45-54	-		0.60	0.54-0.65
	55-64	-		0.45	0.40-0.50
	65+	-		0.24	0.21-0.26
Age x autism	25-34 (ref)	-		-	
	35-44	-		0.83	0.72-0.96
	45-54	-		0.59	0.50-0.68
	55-64	-		0.49	0.41-0.59
	65+	-		0.44	0.36-0.53
Constant		0.08		0.08	

For peer review only

For peer review only - http://bmjopen.bmj.com/site/about/guidelines.xhtml

STROBE 2007 (v4) Statement—Checklist of items that should be included in reports of *cross-sectional studies*

Section/Topic	Item #	Recommendation	Reported on page #
**Title and abstract**	1	(*a*) Indicate the study's design with a commonly used term in the title or the abstract	Page 1
		(*b*) Provide in the abstract an informative and balanced summary of what was done and what was found	Page 2
**Introduction**			
Background/rationale	2	Explain the scientific background and rationale for the investigation being reported	Page 2<br>Section: Introduction
Objectives	3	State specific objectives, including any prespecified hypotheses	Page 2<br>Section: Introduction
**Methods**			
Study design	4	Present key elements of study design early in the paper	Page 2-4<br>Section: Methods/Census process and key variables
Setting	5	Describe the setting, locations, and relevant dates, including periods of recruitment, exposure, follow-up, and data collection	Page 2-4<br>Section: Methods/Census process and key variables
Participants	6	(*a*) Give the eligibility criteria, and the sources and methods of selection of participants	Page 2-4<br>Section: Methods/Census process and key variables
Variables	7	Clearly define all outcomes, exposures, predictors, potential confounders, and effect modifiers. Give diagnostic criteria, if applicable	Page 2-4<br>Section: Methods
Data sources/ measurement	8*	For each variable of interest, give sources of data and details of methods of assessment (measurement). Describe comparability of assessment methods if there is more than one group	Page 2-4<br>Section: Methods/Census process and key variables
Bias	9	Describe any efforts to address potential sources of bias	Page 2-4<br>Section: Methods
Study size	10	Explain how the study size was arrived at	Page 2-4<br>Section: Methods/Census process and key variables

For peer review only - http://bmjopen.bmj.com/site/about/guidelines.xhtml

Quantitative variables	11	Explain how quantitative variables were handled in the analyses. If applicable, describe which groupings were chosen and why	Page 4<br>Section: Methods/Data analysis
Statistical methods	12	(*a*) Describe all statistical methods, including those used to control for confounding	Page 4<br>Section: Methods/Data analysis
		(*b*) Describe any methods used to examine subgroups and interactions	Page 4<br>Section: Methods/Data analysis
		(*c*) Explain how missing data were addressed	Page 2-4<br>Section: Methods/Census process and key variables
		(*d*) If applicable, describe analytical methods taking account of sampling strategy	N/A
		(*e*) Describe any sensitivity analyses	N/A
**Results**			
Participants	13*	(a) Report numbers of individuals at each stage of study—eg numbers potentially eligible, examined for eligibility, confirmed eligible, included in the study, completing follow-up, and analysed	Page 5<br>Section: Results
		(b) Give reasons for non-participation at each stage	N/A
		(c) Consider use of a flow diagram	N/A
Descriptive data	14*	(a) Give characteristics of study participants (eg demographic, clinical, social) and information on exposures and potential confounders	Page 5<br>Section: Results
		(b) Indicate number of participants with missing data for each variable of interest	Page 2-4<br>Section: Methods/Census process and key variables
Outcome data	15*	Report numbers of outcome events or summary measures	N/A
Main results	16	(*a*) Give unadjusted estimates and, if applicable, confounder-adjusted estimates and their precision (e.g., 95% confidence interval). Make clear which confounders were adjusted for and why they were included	Pages 5-6<br>Section: Results<br>Pages 10-13 Tables 1-4
		(*b*) Report category boundaries when continuous variables were categorized	Page 4<br>Section: Methods/Data analysis

For peer review only - http://bmjopen.bmj.com/site/about/guidelines.xhtml

			Pages 10-13 Tables 1-4
		(*c*) If relevant, consider translating estimates of relative risk into absolute risk for a meaningful time period	N/A
Other analyses	17	Report other analyses done—eg analyses of subgroups and interactions, and sensitivity analyses	N/A
**Discussion**			
Key results	18	Summarise key results with reference to study objectives	Page 6<br>Section: Discussion/ Principal findings and interpretation; Comparison with previous literature
Limitations	19	Discuss limitations of the study, taking into account sources of potential bias or imprecision. Discuss both direction and magnitude of any potential bias	Page 6-7<br>Section: Strengths and limitations
Interpretation	20	Give a cautious overall interpretation of results considering objectives, limitations, multiplicity of analyses, results from similar studies, and other relevant evidence	Page 6<br>Section: Discussion/ Principal findings and interpretation; Comparison with previous literature
Generalisability	21	Discuss the generalisability (external validity) of the study results	Page 7<br>Section: Implications
**Other information**			
Funding	22	Give the source of funding and the role of the funders for the present study and, if applicable, for the original study on which the present article is based	Page 8<br>Section: Funding

*Give information separately for exposed and unexposed groups.

Note: An Explanation and Elaboration article discusses each checklist item and gives methodological background and published examples of transparent reporting. The STROBE checklist is best used in conjunction with this article (freely available on the Web sites of PLoS Medicine at http://www.plosmedicine.org/, Annals of Internal Medicine at http://www.annals.org/, and Epidemiology at http://www.epidem.com/). Information on the STROBE Initiative is available at www.strobe-statement.org.

For peer review only
